# Targeting DOT1L Epigenetic Moonlighting in MLL-Rearranged Leukemia

**DOI:** 10.3390/cells15151399

**Published:** 2026-08-03

**Authors:** Dikshat Gopal Gupta, Monika Gupta, Ahmad Hasan Othman, Uzer Abdulaziz Memon, Gary E. Schiltz, Sarki A. Abdulkadir

**Affiliations:** 1 Department of Urology, The Robert H. Lurie Comprehensive Cancer Center, Northwestern University Feinberg School of Medicine, Chicago, IL 60611, USA; ahmadothmanphd@gmail.com (A.H.O.); sarki.abdulkadir@northwestern.edu (S.A.A.); 2 Department of Neurology, Post Graduate Institute of Medical Education and Research, Chandigarh 160012, India; drmonikarawla07@gmail.com; 3 NHL Municipal Medical College, Ahmedabad 380006, India; uzer.a.memon@gmail.com; 4 Department of Chemistry, Northwestern University, Evanston, IL 60208, USA; gary-schiltz@northwestern.edu; 5 The Robert H. Lurie Comprehensive Cancer Center, Northwestern University Feinberg School of Medicine, Chicago, IL 60611, USA; 6 Department of Pathology, Northwestern University Feinberg School of Medicine, Chicago, IL 60611, USA

**Keywords:** acute lymphoblastic leukemia, acute myeloid leukemia, DOT1L, *KM2TA*-rearranged leukemia, PROTACs, small molecule inhibitors

## Abstract

**Highlights:**

**What are the main findings?**

- While the DOT1L catalytic inhibitor pinometostat showed only modest clinical activity in KMT2A-rearranged leukemia due to poor pharmacokinetics and an inability to block DOT1L-methyltransferase-independent functions, recent studies have confirmed that DOT1L drives leukemogenesis through “epigenetic moonlighting” including transcriptional regulation, histone chaperone activity, and scaffolding that operate independently of its H3K79 methyltransferase function.
- To overcome these limitations, first-in-class VHL-recruiting PROTAC degraders (DOT1L705, DOT1L808, and MS2133) have been developed that selectively eliminate both the catalytic and methyltransferase-independent oncogenic functions of DOT1L via the ubiquitin–proteasome system.
- In preclinical KMT2A-rearranged leukemia models, targeted degradation of DOT1L with PROTACs achieves potent antileukemic efficacy, with lead compound DOT1L808 demonstrating complete tumor regression in orthotopic mouse studies without observable toxicity.
- Furthermore, combining DOT1L degradation with Menin inhibition creates a compelling dual strategy that simultaneously disrupts the N-terminal Menin–MLL interaction while eradicating all DOT1L functions within the MLL-fused complex.
- Despite promising preclinical data, DOT1L-targeting PROTACs have not yet entered clinical trials, and substantial challenges including pharmacokinetic optimization, toxicity management, and clinical validation remain to be addressed.

**What are the implications of the main findings?**

- The failure of catalytic inhibition, coupled with the discovery of methyltransferase-independent functions, establishes that blocking enzymatic activity alone is insufficient, necessitating a paradigm shift toward total protein degradation for therapeutic success in this high-risk leukemia subtype.
- The successful development of selective DOT1L PROTAC degraders provides a validated approach and a clinically translatable strategy to fully eliminate DOT1L’s oncogenic activity, potentially redefining the standard of care for KMT2A-rearranged leukemia patients who currently lack effective targeted therapies.
- The robust in vivo efficacy and favorable safety profile of DOT1L808 support its advancement toward clinical trials, offering a new therapeutic avenue where catalytic inhibitors have fallen short.
- Finally, the synergistic rationale for combining DOT1L degradation with Menin inhibition presents a promising dual-hit approach that may prevent resistance mechanisms and enhance durable remission rates by attacking two critical nodes of the MLL oncogenic complex simultaneously.
- However, the clinical translation of PROTACs faces substantial challenges, including suboptimal pharmacokinetics, potential toxicity, and the need for rigorous clinical validation.

**Abstract:**

KMT2A-rearranged (MLL-r) leukemias are highly aggressive hematological malignancies that require improved targeted therapies. DOT1L (histone H3K79 methyltransferase) functions as a critical oncogenic driver and represents an important therapeutic target in these high-risk leukemias. However, clinical responses to the first-in-class DOT1L inhibitor pinometostat (EPZ5676) have been modest, attributed to suboptimal pharmacokinetics and, more fundamentally, to the recognition that DOT1L possesses methyltransferase-independent functions that evade catalytic inhibition. This highlights the need for strategies that abrogate the full spectrum of DOT1L activity to effectively treat these high-risk leukemias. Proteolysis-targeting chimeras (PROTACs), which induce selective degradation of the DOT1L protein rather than inhibiting its catalytic activity, have therefore emerged as a promising approach. Notably, VHL-recruiting DOT1L PROTACs, such as DOT1L808, have demonstrated improved pharmacokinetic profiles and potent antileukemic activity in preclinical in vivo models. However, these findings remain preclinical, and significant challenges including oral bioavailability, potential toxicity, and lack of clinical validation must be addressed before clinical translation. In this review, we provide an overview of the evolving understanding of the biology of DOT1L, discuss existing MLL small molecule therapies, and evaluate current advances in therapeutically targeting *DOT1L*, with particular focus on the targeted degradation of DOT1L as a promising therapeutic strategy for high-risk KMT2A-r leukemia.

## 1. MLL-Rearranged Leukemia: Breaking the Key, Not the Lock

Chromosomal rearrangements of the *KMT2A* (MLL) gene at chromosome 11q23 define a highly aggressive genetic subtype of acute leukemia that is associated with poor clinical outcomes in both pediatric and adult patients [1,2,3,4,5]. *KMT2A*-r leukemias predominantly affect infants under 1 year of age and represent a significant proportion of pediatric acute lymphoblastic leukemia (ALL) (~80%) and acute myeloid leukemia (AML) (35–50%), with a 5-year event-free survival of 46% [2,3,6,7,8,9,10,11]. In adults, the incidence is lower (~5% of ALL and 5–10% of AML), with comparably poor outcomes [5,12,13,14,15,16]. Clinically, this subtype is marked by its aggressive nature, early relapses, hyperleukocytosis, and frequent central nervous system (CNS) involvement [7,17,18].

**Figure 1:**
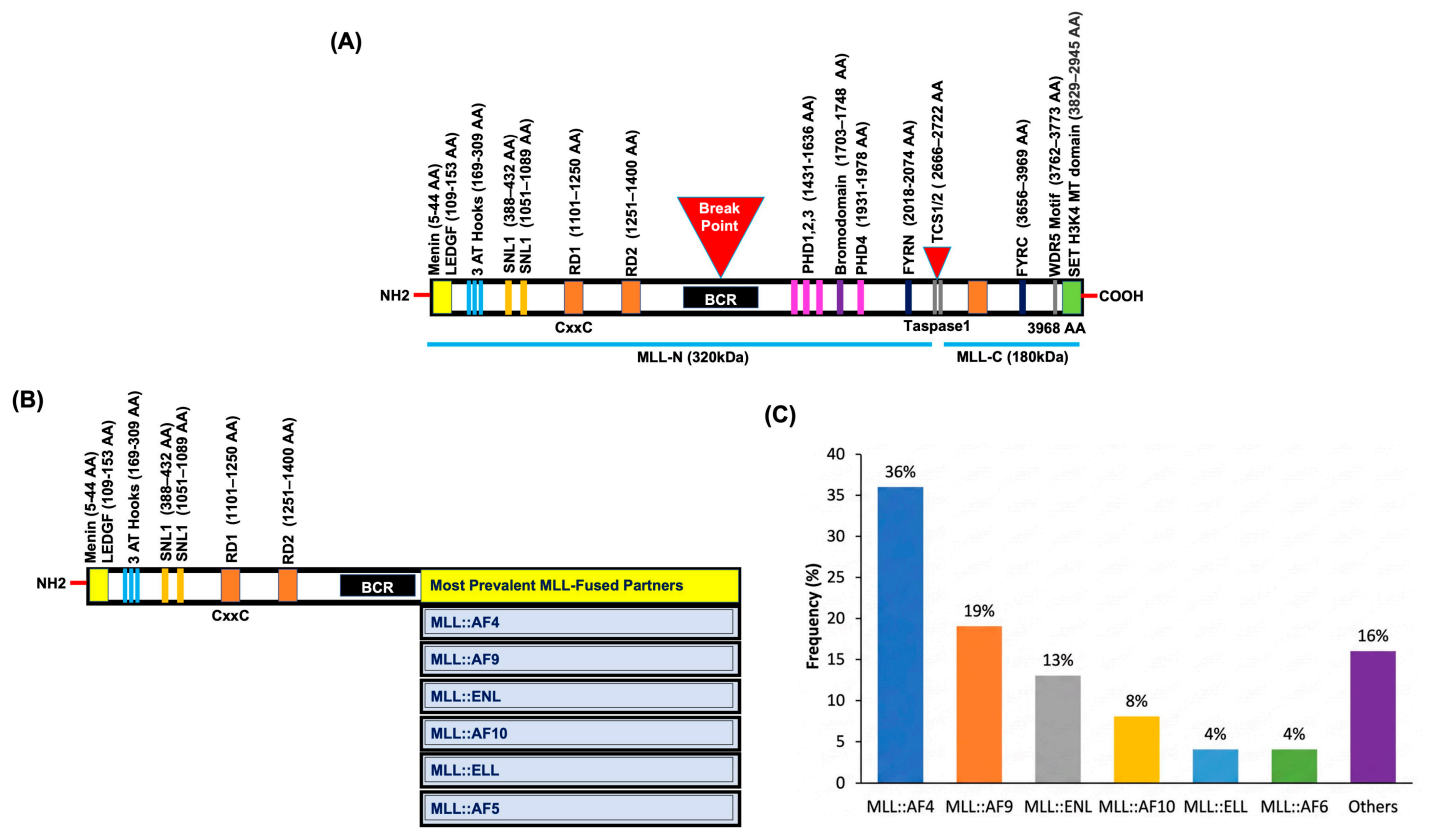
(**A**) Overview of MLL gene structure. N-terminal features include the Menin binding domain connecting LEDGF with MLL1, three AT hooks motifs for DNA binding, and sub-nuclear localization peptides (SNL1, SNL2). Repression domains (RD1, RD2) interact with polycomb group proteins and histone deacetylases. The middle section showcases four PHD zinc fingers, a bromodomain, and a transactivation domain. Nuclear CYP33 regulates HOX cluster genes, and taspase1 cleaves MLL into MLL-N and MLL-C. The C-terminal comprises Win (WDR5) and the SET domain for H3K4 methylation. (**B**,**C**) Common fusion partners of MLL gene. The N-terminal region of the MLL gene, which harbors the DNA-binding domain, undergoes chromosomal translocations with over 80 different in-frame fusion partners in *KMT2A*-rearranged leukemia. The most prevalent partners include *MLL::AF4*, *MLL::AF9*, *MLL::ENL*, *MLL::AF10*, *MLL::ELL*, and *MLL::AF6*, each representing a distinct translocation event that contributes to the molecular diversity observed in *KMT2A*-r leukemia. Panel C shows the distribution of common MLL fusions in acute leukemias (including both ALL and AML), adapted from Meyer et al., 2017 [12,13].

The pathobiology of *KMT2A*-r leukemia is driven by in-frame fusions of the MLL gene with >80 fusion partners [5,13,19,20,21]. The most prevalent partners, including *MLL::AF4*, *MLL::AF9*, *MLL::ENL*, *MLL::AF10*, *MLL::ELL* and *MLL::AF6*, account for ~80% of cases (Figure 1, Table 1) [7,12,13,22,23,24,25,26,27,28,29,30,31,32,33]. The significance of these in-frame MLL fusions is the aberrant recruitment of the histone methyltransferase *DOT1L* (Disruptor of Telomeric Silencing 1-like). Several partners, such as *AF9*, *ENL*, *AF10*, and *AF17*, are integral components of the DOT1L-containing DotCom complex [34,35], enabling ectopic H3K79 methylation and sustained activation of leukemogenic programs, including *HOXA9* and *MEIS1* [7,20,27,36,37,38]. Beyond DOT1L, the *KMT2A* fusion complex comprises several additional essential transcriptional factors that collectively drive leukemogenesis [27,34,39]. Menin (MEN1) serves as a critical scaffold protein that binds the N-terminus of *KMT2A* via the Menin-binding domain (MBD), anchoring the fusion complex to chromatin and enabling stable formation of the oncogenic transcriptional machinery. The interaction between Menin and *KMT2A* is essential for maintaining leukemic transformation, and its disruption has emerged as a highly promising therapeutic strategy [39,40,41,42,43,44,45,46,47,48,49,50].

Additionally, most *KMT2A* fusion proteins recruit the Super Elongation Complex (SEC), a multi-protein complex composed of ELL family members (*ELL1–3*), *EAF1–2*, and the positive transcription elongation factor b (*P-TEFb*) [51,52]. SEC promotes transcriptional elongation through phosphorylation of RNA Polymerase II, thereby driving sustained expression of leukemogenic target genes including *HOXA9* and *MEIS1* [53,54,55]. Many of the most frequent *KMT2A* fusion partners, including *AF4*, *AF9*, *ENL*, and *ELL*, are integral components of the SEC or the related AEP complex (AF4 family/ENL family/P-TEFb), which similarly facilitates oncogenic transcription [56,57].

Furthermore, the *KMT2A* fusion complex physically interacts with several histone acetyltransferases (HATs), including MOZ/MORF, HBO1, and EP300/CREBBP, which effect MLL–MENIN-dependent gene activation through chromatin remodeling [39,46,58]. Other components such as the PAF complex and LEDGF also contribute to chromatin targeting and transcriptional regulation. This multi-component complex comprising Menin (scaffolding), SEC/AEP (transcriptional elongation), DOT1L (H3K79 methylation), and HATs (chromatin remodeling) collectively drives the aberrant transcriptional program characteristic of *KMT2A*-rearranged leukemia [27,46,49,59]. While DOT1L plays a central role as the enzymatic effector of H3K79 methylation, therapeutic targeting of other complex components, particularly Menin, has emerged as a complementary and clinically promising strategy. This mechanism establishes DOT1L as an oncogenic driver in *KMT2A*-r leukemia [20,35,60,61]. Beyond hematological malignancies, DOT1L overexpression has also been implicated in solid tumors, including prostate and breast cancer, where it correlates with poor prognosis [62,63,64,65].

**Table 1:** Incidence of MLL-associated fusions in *KMT2A*-rearranged leukemia.

MLL-Fusions	Disease	Age Wise Distribution			Overall Incidence Range	Reference
		Infant (<1 Year)	Pediatric (>1–18 Years)	Adult (>18 Years)		
*MLL-AF4*	ALL	14.41	5.92	14.15	5–65%	[12,13,14,15,19,22,23,24,26,66]
	AML	0.17	0.12	0.12	0.42–1.7%	
*MLL-AF9*	ALL	4.81	2.38	0.38	6.9–9%	[12,13,14,26]
	AML	1.70	5.62	3.83	2.6–33.5%	
*MLL-ENL*	ALL	6.56	2.38	2.13	0.4–11.08%	[12,13,14,26,67]
	AML	0.08	0.89	0.59	1.4–6.5%	
*MLL-AF10*	ALL	1.66	0.51	0.04	1.6–2.5%	[12,13,14,26]
	AML	1.83	2.81	1.40	2.0–14.5%	
*MLL-ELL*	ALL	0	0	0.04	0.04–1%	[12,13,14,26,67]
	AML	1.02	1.02	1.91	0.87–12%	
*MLL-AF6*	ALL	0.04	0.68	0.38	0.75–2.19%	[12,13,14,15,26,67]
	AML	0.08	1,19	1.62	2.90–12%	

Values in columns 3–5 represent the percentage distribution of each fusion type within the specified age group among *KMT2A*-rearranged leukemia cases. ALL = acute lymphoblastic leukemia, AML = acute myeloid leukemia. MLL refers to the historical gene name for *KMT2A*. Incidence data compiled from Meyer et al., 2013, 2018 and Chen et al., 2021 [12,13,26].

Given its critical oncogenic role, DOT1L has emerged as a key therapeutic target in *KMT2A*-r leukemia [3,4,68,69]. The first-in-class inhibitor EPZ5676 (pinometostat) demonstrated proof-of-concept but limited clinical efficacy [3,36,70]. Mechanistically, this limitation reflects both suboptimal pharmacokinetics and, more fundamentally, the recognition that DOT1L exerts methyltransferase-independent functions in gene regulation and tumorigenesis [35,36,71,72]. Consistently, genetic loss of DOT1L disrupts transcriptional elongation and differentiation, whereas catalytic inactivation does not fully recapitulate these effects, indicating a critical non-catalytic scaffolding role [35,73]. These findings highlight the need for strategies that abrogate all DOT1L functions. Proteolysis-targeting chimeras (PROTACs) have emerged as a powerful technology to selectively degrade target proteins, thereby abrogating both their enzymatic and non-enzymatic functions and producing potent anti-tumor effects in vivo [37,74,75,76,77].

To address this therapeutic challenge, our group and Yim et al. have developed stable VHL-recruiting PROTACs targeting DOT1L. Our degraders, DOT1L705 (DC50 = 0.33 μM) and DOT1L808 (DC50 = 5 nM), potently and selectively degrade DOT1L via the ubiquitin–proteasome system. Notably, DOT1L705 retains activity against Menin inhibitor-resistant cells, and DOT1L808 achieves complete tumor regression in an orthotopic *KMT2A*-r leukemia model without overt toxicity [78]. Yim et al. identified the first-in-class DOT1L degrader, MS2133, which similarly induces potent and selective degradation [79]. Critically, they demonstrated its ability to effectively inhibit *KMT2A*-r leukemia cell growth while sparing normal cells. Targeted degradation of DOT1L overcomes the fundamental limitations of catalytic inhibition and enables superior pharmacological control, making clear that effective intervention requires breaking the key, not simply blocking the lock, in *KM2TA*-r leukemias.

## 2. DOT1L Structure and Molecular Hijacking in *KMT2A*-r Leukemia

**Figure 2:**
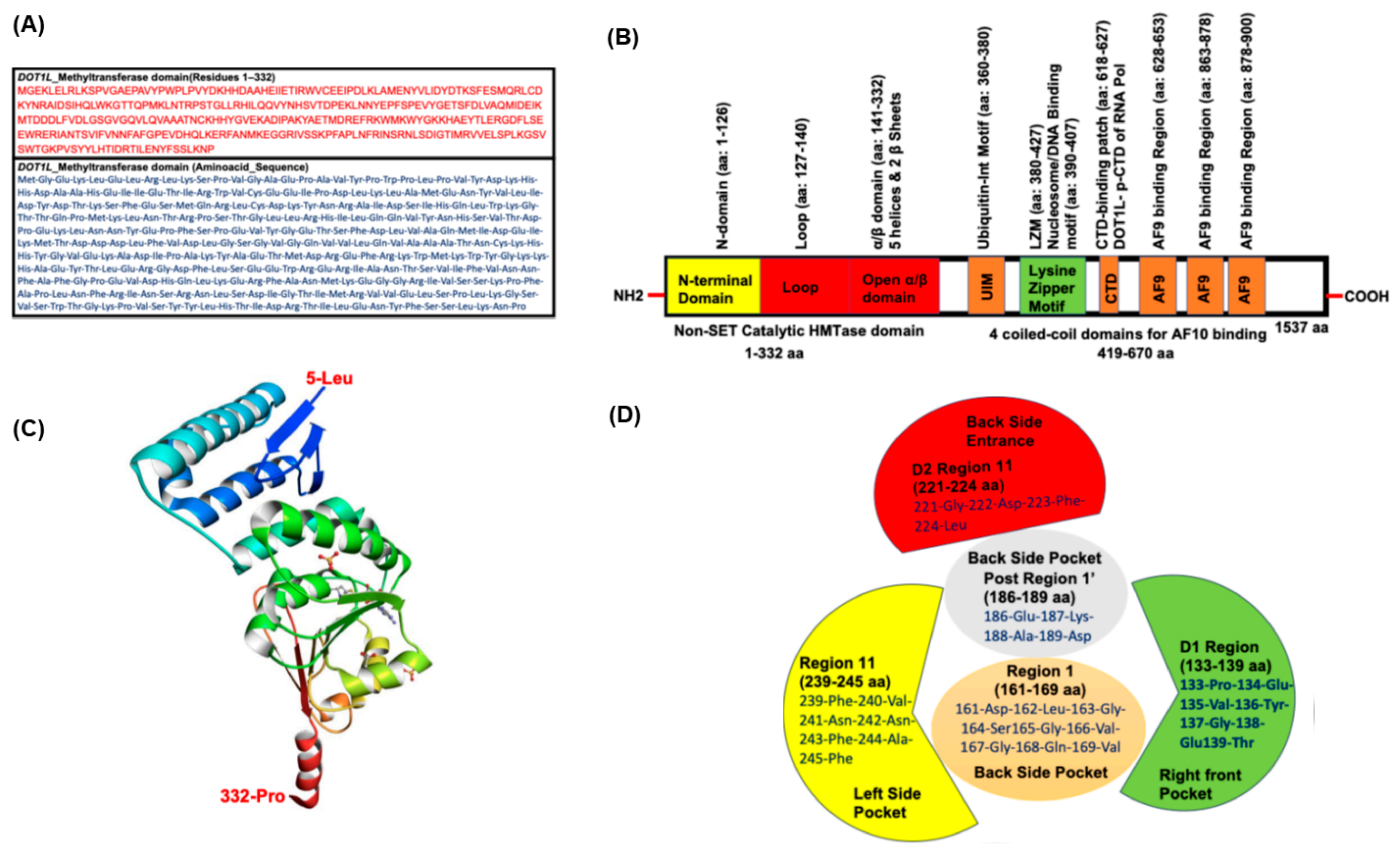
(**A**) Structure of human *DOT1L* and N-terminal catalytic non-SET domain. DOT1L amino acids 1 to 332, housing the histone methyltransferase (HMTase) catalytic non-SET domain. The nucleotide and amino acid sequences of this crucial 1–332 region are depicted, providing insight into the composition of the DOT1L catalytic domain. (**B**) The full-length human DOT1L protein, consisting of 1537 amino acids (aa). The N-terminal region spans from amino acids 1-332, housing the histone methyltransferase (HMTase) catalytic non-SET domain critical for DOT1L’s enzymatic activity. The extensive C-terminal segment follows, featuring three *AF9* binding motif sites, along with binding sites for *AF10* and *ENL*. This detailed depiction offers a comprehensive overview of the domain organization within the C-terminal region, highlighting key interaction sites that play crucial roles in DOT1L’s molecular function. (**C**) Crystal structure of the DOT1L methyltransferase domain, highlighting its overall fold and functional regions including N domains, a protein flexible loop, and an open α/β domain containing helices and β hairpins. The C-terminal domain is depicted with an open α/β structure, featuring β sheets and α helices, with αH unmodeled due to weak electron density. (**D**) SAM binding pocket architecture in DOT1L. This figure details the SAM (S-adenosylmethionine) binding pocket of DOT1L, characterized by five discrete protein segments. The D1 region contributes to the right front pocket, while the backside pocket is shaped by region I and post region I. Additionally, the backside entrance is governed by D2, and the left side pocket is defined by Region II.

**Figure 3:**
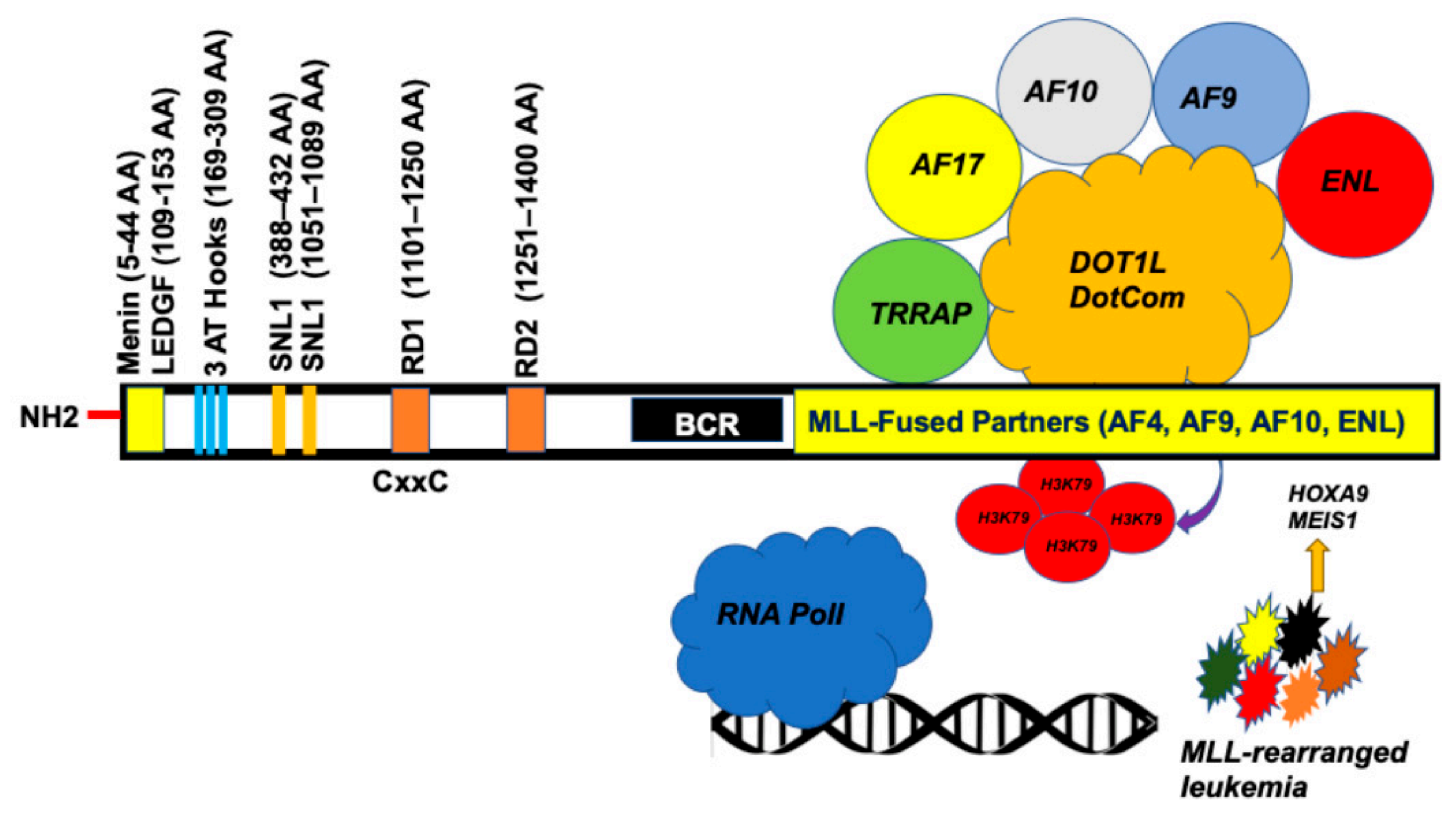
DOT1L interaction and aberrant methylation in *KMT2A*-rearranged leukemia. This figure illustrates the crucial events in *KMT2A*-rearranged leukemia, where DOT1L is sequestered from the DotCom complex by various fusion partners, such as *AF10*, *AF9*, *ENL*, *AF17*, and *TRRAP*. This aberrant interaction results in DOT1L catalyzing abnormal H3K79 methylation on MLL-specific genes, including the *HOXA9* cluster and *MEIS1*, ultimately contributing to the pathogenesis of leukemia.

*DOT1L* was originally discovered in Saccharomyces cerevisiae as a disruptor of telomeric silencing and is an evolutionarily conserved histone methyltransferase from yeast to humans that uniquely lacks a SET domain. *DOT1L* is the only histone methyltransferase that transfers S-methyl from (S)-adenosyl-l-methionine (SAM) to the H3K79 (NH3 group), producing a methylated lysine residue 79 (H3K79) and (S)-adenosyl-l-homocysteine (SAH) allowing for mono, di, and tri-methylation of histone H3 lysine 79 (H3K79) within the nucleosome core, a modification associated with active transcription [80,81,82,83,84,85,86,87,88]. Beyond its enzymatic function, DOT1L regulates diverse biological processes, including transcriptional elongation, cell cycle progression, DNA damage repair, and embryonic development, with constitutive loss resulting in embryonic lethality [89,90,91,92,93,94]. The structure of DOT1L is well-studied and understood, and highly conserved from yeast to humans, as shown in Figure 2 [80,81,82,95,96,97]. Functionally, DOT1L operates within the multi-subunit DotCom complex, where it interacts with proteins such as *AF9*, *ENL*, *AF10*, *AF17*, and *TRRAP* [34,35,80]. These interactions facilitate chromatin targeting, with *AF9* and *ENL* recognizing acetylated histones and AF10 enhancing higher-order H3K79 methylation. In the context of *KMT2A*-rearranged leukemia, aberrant recruitment of DOT1L-containing complexes by MLL fusion proteins drives H3K79 hypermethylation and sustained transcription of leukemogenic genes, including HOXA9 and MEIS1 to drive leukemogenesis, as shown in Figure 3 [55,98,99].

## 3. Epigenetic Moonlighting of DOT1L Methyltransferase

**Figure 4:**
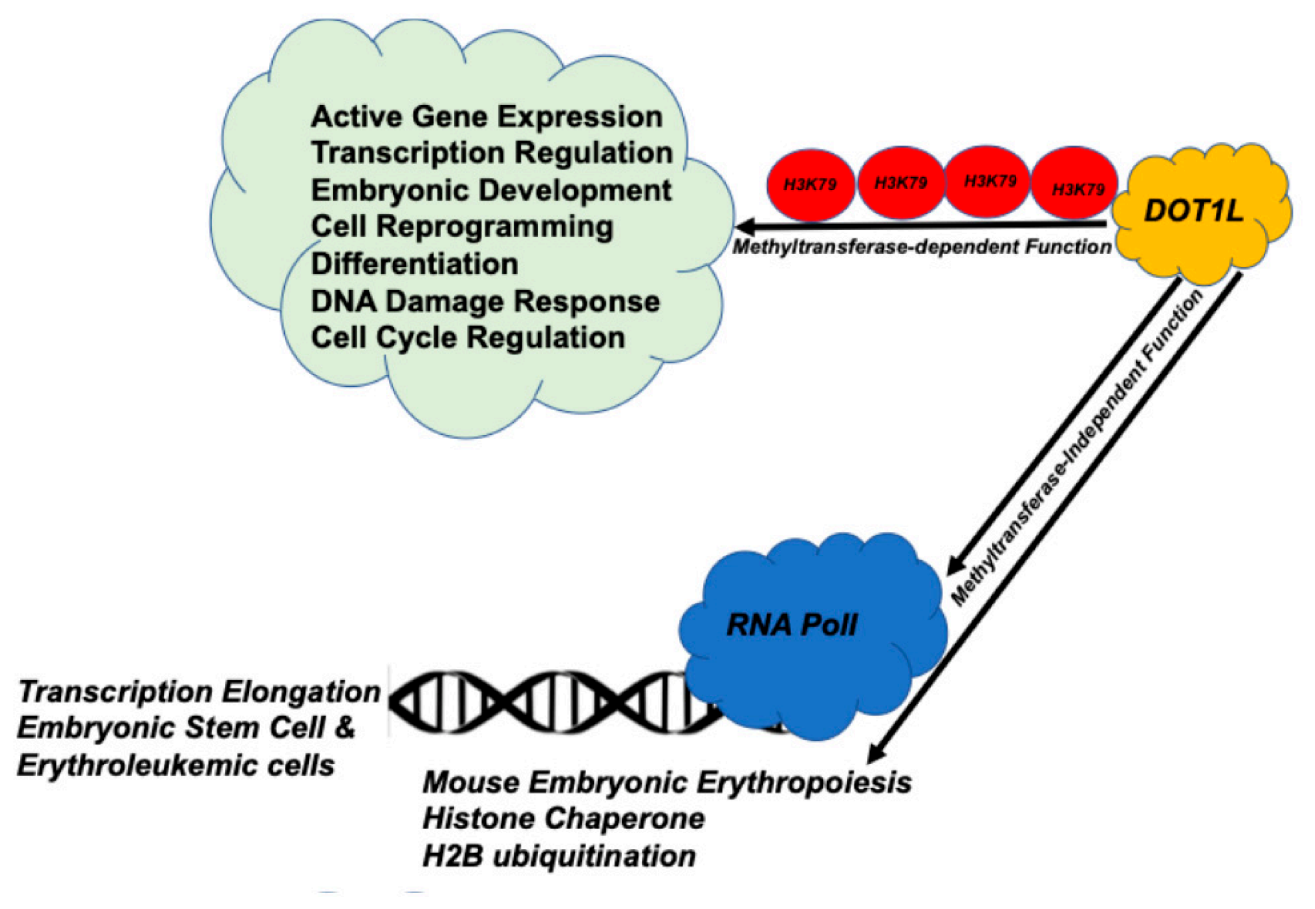
Epigenetic Moonlighting of DOT1L. DOT1L canonical (catalytic methyltransferase-dependent functions) and non-canonical (methyltransferase-independent functions).

Epigenetic moonlighting refers to the broader concept that chromatin regulators perform functions beyond their canonical enzymatic activities. Recent studies have revealed epigenetic moonlighting functions of DOT1L, in which the DOT1L performs methyltransferase-independent functions independent of its catalytic methyltransferase activity and H3K79 methylation. Methyltransferase-independent activities have been observed across multiple model systems, including human erythroleukemia cells, embryonic stem cells (ESCs), and mouse primitive erythropoiesis, as shown in Figure 4 [35,56,73,100]. However, it is important to distinguish that direct evidence for these functions in *KMT2A*-rearranged leukemia currently comes primarily from genetic depletion studies (Cao et al., 2020) [35], while findings from ESCs, erythroid, and yeast models establish mechanistic principles that require direct validation in leukemia, as shown in Table 2.

Zhao et al. (2023) [101] demonstrated a methyltransferase-independent transcriptional repressive role of DOT1L in ESCs, mediated through histone H1 chaperoning in cooperation with nucleophosmin (NPM1). Through this mechanism, DOT1L represses endogenous retroviruses, particularly MERVL elements. Although DOT1L is classically associated with H3K79 methylation and transcriptional activation, this study revealed that DOT1L can also function as a transcriptional repressor in pluripotent cells through chromatin-structural mechanisms [101]. Importantly, this finding in ESCs establishes the principle of methyltransferase-independent chromatin regulation but has not been directly validated in *KMT2A*-rearranged leukemias.

Evidence for methyltransferase-independent functions has also emerged from developmental studies. Malcom et al. (2021) [100] demonstrated that DOT1L methyltransferase-mutant (MM) mouse embryos, harboring a catalytic Asp241Ala mutation, displayed markedly different phenotypes compared with DOT1L knockout embryos. Whereas DOT1L knockout embryos exhibit lethal anemia during embryonic erythropoiesis, the methyltransferase-mutant embryos showed no overt anemia and appeared largely comparable to wild-type embryos, suggesting that DOT1L possesses critical methyltransferase-independent functions during primitive erythropoiesis [100]. While this establishes the moonlighting concept in development, the relevance of this specific function to leukemogenesis requires direct testing in *KMT2A*-r leukemia models.

Additional studies have implicated DOT1L in transcriptional regulation independent of its catalytic activity. Wu et al. (2021) [73] reported that DOT1L promotes transcription initiation in erythroleukemia cells by recruiting the *TFIID* transcription complex and enhancing H2B monoubiquitination through limiting deubiquitination vis the SAGA complex. Although DOT1L depletion reduced global chromatin occupancy of transcriptional machinery, it did not significantly alter RNA polymerase II elongation rates, indicating that DOT1L primarily regulates transcription initiation rather than elongation, likely through catalytic-independent mechanisms [73]. This study provides mechanistic evidence in erythroleukemia cells but has not been validated in *KMT2A*-r leukemia, where the relevant transcriptional programs and DOT1L functions may differ.

Similarly, Cao et al. (2020) [35] showed that while DOT1L is required for maintaining proper neural progenitor differentiation in ESCs, catalytic inactivation of DOT1L (mutations in Gly163 and Ser164 within the catalytic pocket) did not significantly impair transcriptional elongation. These findings further support the concept that DOT1L contributes to transcriptional regulation in ESCs through functions that are partially independent of H3K79 methylation [35].

Studies in yeast have also provided mechanistic insights into methyltransferase-independent activities of Dot1. Van WT et al. (2018) demonstrated that the N-terminal region of yeast Dot1 promotes H2B ubiquitination independent of H3K79 methylation, revealing a bidirectional crosstalk between Dot1 and H2B ubiquitination pathways and suggesting broader chromatin regulatory functions beyond its catalytic activity [102]. In addition, Lee et al. (2018) reported that yeast Dot1 can function as a histone chaperone, regulating chromatin nucleosome dynamics through histone exchange and thereby influencing transcriptional regulation independently of its methyltransferase function [103].

Collectively, these findings demonstrate that DOT1L acts not only as a histone methyltransferase but also as a multifunctional chromatin regulator, participating in transcription initiation, chromatin remodeling, histone chaperone activity, and regulation of H2B ubiquitination. These methyltransferase-independent activities of DOT1L expand its role in chromatin biology beyond H3K79 methylation.

## 4. Histone Methyltransferase DOT1L Inhibitors

The unique non-SET pocket of DOT1L has made it an attractive target, and extensive research has been carried out into targeting small-molecule inhibitors to block or displace SAM from its binding pocket, offering a promising therapeutic strategy that yielded the first generation of inhibitors. [3,4,36,80,81,104]. The classical SAM-competitive inhibitor, EPZ004777, demonstrated proof-of-concept with potent, selective DOT1L inhibition (IC50 = 0.3 nM) and anti-proliferative effects in *KMT2A*-r cell lines. However, it also revealed the first major hurdle, poor drug-like properties; its size and hydrophobicity resulted in low permeability and solubility, limiting its clinical potential [4,105]. Subsequently, by optimizing the EPZ004777 scaffold, EPZ-5676 (pinometostat) was developed to occupy the SAM-binding pocket with enhanced drug-like properties, exhibiting an IC50 of 3.5 nM and robust preclinical efficacy in both in vitro and in vivo *KMT2A*-r leukemia models, including sustained growth inhibition of MV4-11 cells and dose-dependent antitumor activity with low recurrence risk in rat xenografts [3,80,106,107].

In parallel, extensive efforts have been made to expand the repertoire of potent DOT1L inhibitors beyond nucleoside mimetics using a range of high-throughput strategies, including mechanism-guided inhibitor discovery, surface plasmon resonance (SPR), virtual screening (VS), high-throughput screening (HTS), and structure-based design (SBD). These approaches have involved structural modification of SAM, SAH, and non-SAH analogs [3,4,69,105,108,109,110,111,112,113,114,115]. Beyond nucleoside analogs, structurally diverse inhibitors have been identified. DC-L115, discovered through structure-based virtual screening, is a non-nucleoside inhibitor (IC50 = 1.5 μM) selectively cytotoxic to *KMT2A*-r leukemic cells without affecting non-malignant or solid tumor cell lines [109]. *FED1*, an adenine derivative modified from EPZ004777, achieves potent DOT1L inhibition (IC50 = 2.9 nM) through nitrogen substitution at the 7-position [105,116]. Natural product derivatives have also shown promise: Massonianoside B competitively binds DOT1L at the SAM site (IC50 = 400 nM), reducing H3K79 methylation and selectively killing MV4-11 cells [111], while selenopsammaplin A analogs, particularly compound 10, demonstrate potent DOT1L inhibition and anticancer activity in metastatic breast cancer models [110].

Continued discovery efforts have yielded additional chemical scaffolds. Compound 6, featuring a [1,2,4]-triazolo-[3,4-b][1,3,4]-thiadiazole core, selectively inhibits *KMT2A*-r cell proliferation and induces apoptosis (IC50 = 21.9 μM) [117]. Compounds 3 and 9 were identified through integrated AlphaLISA-based HTS, biochemical validation, and molecular docking [114]. Second-generation inhibitors include compound 25, a non-nucleoside agent with dose-dependent cytotoxicity in murine *MLL-AF9* transformed cells (IC50 = 1.0 μM) [112], and compound 12, developed via structure-based fragmentation, which exhibits exceptional potency (IC50 = 1.2 nM), oral bioavailability, and a novel binding mode [115]. A comprehensive summary of these inhibitors, including their structures and developmental status, is provided in Table 3.

### 4.1. Lessons Learned from Catalytic Inhibitor Failures and Future Development

Despite these intensive medicinal chemistry efforts spanning over a decade and achieving picomolar potency, pinometostat demonstrated only modest clinical activity [3], and this translational gap highlights six critical lessons: (1) biochemical potency does not guarantee clinical efficacy, as pharmacological limitations including poor oral bioavailability, rapid clearance, and continuous IV infusion requirements restricted adequate drug exposure; (2) pharmacokinetic properties are as important as target engagement, and suboptimal drug distribution may have limited efficacy in the bone marrow and CNS; (3) catalytic inhibition cannot address DOT1L’s non-enzymatic functions, and the persistence of methyltransferase-independent scaffolding and chromatin regulatory activities allows leukemic cells to maintain oncogenic programs; (4) predictive biomarkers are essential for patient selection, but their absence resulted in the inclusion of heterogeneous patient populations with varying degrees of DOT1L dependency; (5) combination strategies may be required for durable responses, as single-agent inhibition may be insufficient to overcome complex oncogenic networks; and (6) trial design must incorporate efficacy endpoints and biomarker-driven stratification rather than focusing solely on safety and pharmacokinetics. The clinical trials of pinometostat are summarized in Table 4. Additionally, the emergence of resistance through compensatory pathways and epigenetic reprogramming further limited clinical responses. These limitations provide a compelling rationale for targeted protein degradation as a mechanistically superior strategy, and the lessons summarized in Table 5 should inform the clinical development of next-generation DOT1L-targeted therapies, including PROTAC degraders.

### 4.2. Patient Selection, Biomarkers, and Resistance Mechanisms

The modest clinical efficacy of EPZ5676 underscores the need for improved patient stratification, predictive biomarkers, and strategies to overcome resistance [70,106,118]. Patient selection currently relies on *KMT2A* (MLL) rearrangement status; however, accumulating evidence indicates that DOT1L dependency varies among fusion subtypes. Leukemias harboring fusion partners that directly recruit DOT1L (e.g., *AF4*, *AF9*, *ENL*, *AF10*) appear more dependent on DOT1L than those with indirect recruitment mechanisms (e.g., *AF6*, *ELL*) [61,119,120]. Additional biomarkers, including H3K79 methylation, HOXA9/MEIS1 expression, DOT1L protein abundance, and cooperating genomic alterations, may further refine patient selection [121,122]. Resistance to DOT1L inhibitors may arise through epigenetic rewiring, activation of compensatory chromatin regulators (e.g., BRD4 and EZH2), alterations in downstream oncogenic pathways, or pharmacokinetic mechanisms [123,124,125]. Importantly, catalytic inhibition leaves DOT1L’s methyltransferase-independent scaffolding functions intact, allowing leukemic cells to maintain oncogenic transcriptional complexes despite enzymatic blockade [35,73]. By eliminating the entire DOT1L protein, PROTAC degraders simultaneously abolish catalytic activity and non-catalytic scaffolding functions, potentially overcoming a key limitation of catalytic inhibitors. Degradation may also disrupt DOT1L-dependent protein complexes, maintain activity in inhibitor-resistant settings, and produce broader transcriptional reprogramming that reduces compensatory escape mechanisms. Nevertheless, resistance to degraders may emerge through alterations in E3 ligase function, proteasome activity, or drug uptake, emphasizing the importance of biomarker-guided clinical development.

## 5. DOT1L-Degraders/Proteolysis-Targeting Chimeras (PROTACs)

**Figure 5:**
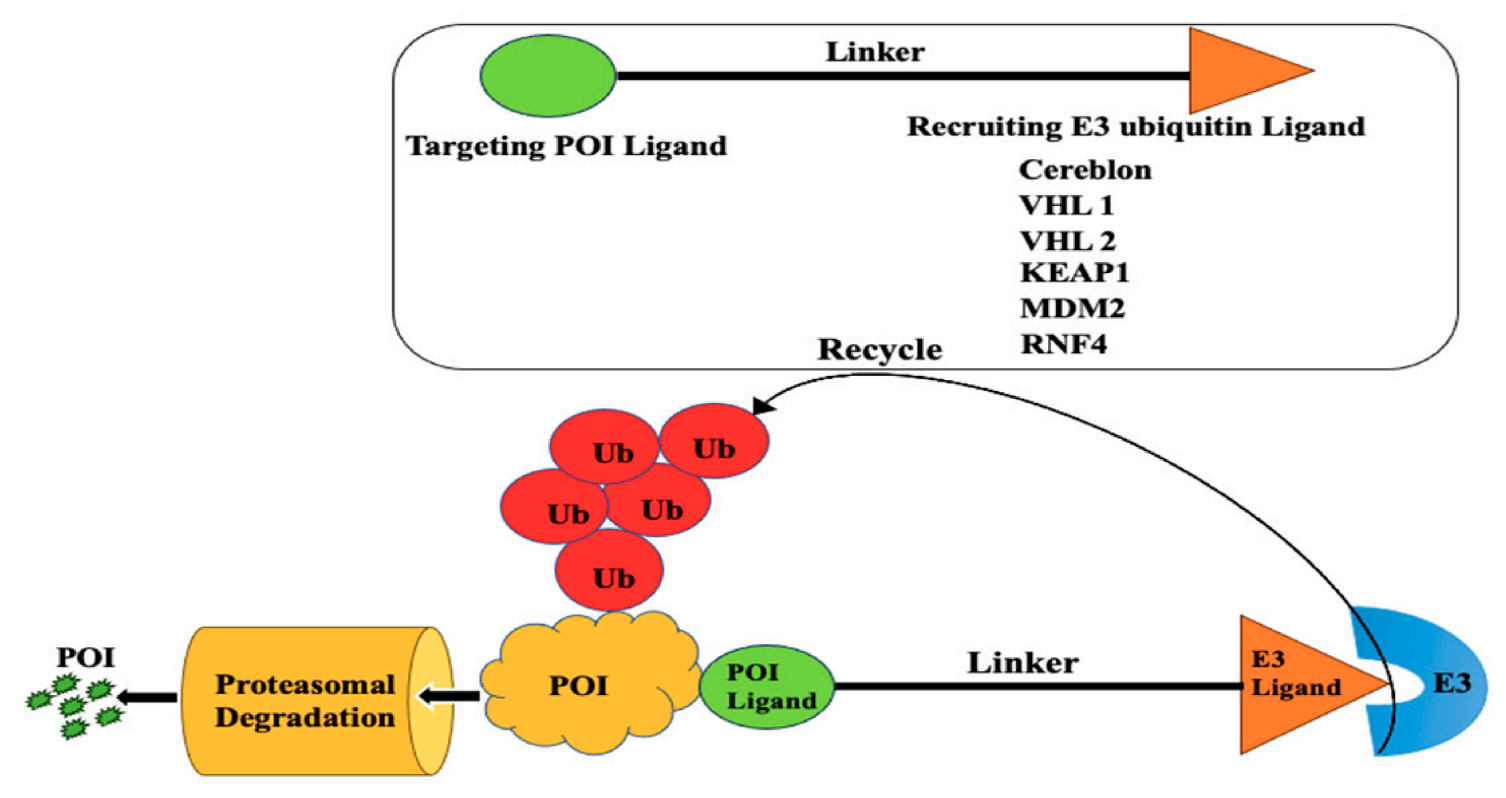
**Basic PROTAC technology**. Schematic diagram showing the key steps: a target protein ligand, a linker, and an E3 ligase ligand form a stable ternary complex, leading to ubiquitination and proteasomal degradation of the target protein. This figure summarizes the core mechanism of PROTAC-mediated targeted protein degradation.

Given the emerging methyltransferase-independent functions of DOT1L and the modest clinical activity of catalytic inhibitors, therapeutic strategies capable of abrogating both methyltransferase-dependent and -independent functions are of considerable interest [35,73]. Recent studies also proposed DOT1L targeted protein degradation via PROTACs by Cao et al. (2020) and Wu A et al. (2021) and could be extremely beneficial for cancer therapeutics/treatments [35,73]. Targeted protein degradation (TPD) via PROTACs has emerged as a powerful drug discovery technology. These bifunctional molecules simultaneously bind a protein of interest (POI) and an E3 ubiquitin ligase, inducing POI ubiquitination and proteasomal degradation while enabling PROTAC recycling for subsequent targeting, as shown in Figure 5. PROTACs offer several advantages over small molecule inhibitors: more robust and sustained functional inhibition, enhanced selectivity through ternary complex formation requirements, and the ability to eliminate both enzymatic and non-enzymatic functions of proteins such as DOT1L [37,74,75,77,126,127,128,129,130,131,132,133,134]. However, these advantages must be weighed against substantial challenges in clinical translation, including suboptimal pharmacokinetic properties, potential on-target and off-target toxicity, and the lack of clinical validation for DOT1L-specific degraders. Consequently, PROTACs have gained increasing recognition as a therapeutic strategy for hematological disorders, with several candidates targeting critical oncogenic proteins advancing to clinical trials, as shown in Table 5.

## 6. Rationale of Designing DOT1L-Targeting PROTAC

In the PROTAC mechanism of action, formation of a stable, high-affinity ternary complex between the POI, PROTAC, and E3 ligase is critical for efficient degradation [134,135]. This is governed by optimal selection of E3 ligands, POI ligands, and linker composition including attachment sites, length, and flexibility which collectively determine ternary complex stability. To date, numerous successful ternary complexes (e.g., POI-PROTAC-VHL/CRBN) have been developed, enabling targeted degradation of previously undruggable proteins. These advancements have progressed to clinical trials, demonstrating their therapeutic promise. Notably, MZ1 (a POI-PROTAC-VHL complex targeting BRD) showed anticancer effects on AML cell lines by selectively degrading BRD2,3 and BRD4 [136,137], while NX-2127 and NX-5948 (POI-PROTAC-CRBN complexes) are under evaluation for relapsed/refractory B cell malignancies (NCT04830137, NCT05131022). Given DOT1L’s methyltransferase-independent functions in *KMT2A*-r leukemias, a potent DOT1L-PROTAC is urgently needed. Despite challenges in forming a stable DOT1L ternary complex, breakthroughs like MZ1, NX-2127, and NX-594,8 which successfully degrade BRD, IKZF1, and BTK, provide a strong rationale that a DOT1L-PROTAC could similarly transform the treatment paradigm for *KMT2A*-rearranged leukemias [136].

To date, two peer-reviewed reports have described PROTACs specifically designed to target and degrade DOT1L. Yim et al. reported MS2133, a VHL-recruiting PROTAC that potently induces concentration and time-dependent DOT1L degradation without affecting mRNA expression, demonstrating selectivity for DOT1L over other methyltransferases and effective growth inhibition of *KMT2A*-r leukemia cells with no toxicity to normal cells, though in vivo and PK studies were not conducted [79]. Subsequently, our group developed novel DOT1L-targeting PROTACs (DOT1L705 and DOT1L808) with DC50 values of 0.33 μM and 5 nM, respectively, which potently and selectively degrade DOT1L via the ubiquitin–proteasome system. These PROTACs effectively inhibit *KMT2A*-r leukemia cell viability, retain activity against Menin inhibitor-resistant cells, and critically, DOT1L808 achieves complete tumor regression in an orthotopic leukemia model without overt toxicity, establishing protein degradation as a promising therapeutic strategy for *KMT2A*-rearranged leukemias by abrogating both enzymatic and non-enzymatic functions of DOT1L [78,79]. A balanced comparison of all three DOT1L degraders is presented in Table 6, highlighting their respective advantages and limitations. MS2133 established the proof-of-concept for DOT1L degradation, while DOT1L808 currently offers the most robust in vivo efficacy data. Each compound has distinct properties that may suit different therapeutic contexts, and further head-to-head studies would be valuable to inform clinical development.

## 7. Synergistic Strategies: Targeting Menin and DOT1L in *KMT2A*-r Leukemia

Menin binds the MLL N-terminus via the first five amino acids, a critical interaction driving gene expression in *KMT2A*-r leukemias [46,138,139,140]. Menin inhibitors have emerged as a promising therapeutic strategy, with DS-1594a, KO-539, DSP-5336, BMF-219, currently in clinical trials, and FDA-approved SNDX-5613, as shown in Table 7. In preclinical investigations, DS-1594a demonstrated 50% greater reduction in proliferative activity than cytarabine in preclinical ALL and AML models, along with enhanced *KMT2A*-r AML differentiation in vitro, leading to an ongoing Phase I trial (NCT04752163) expected to finish in 2030 [48]. KO-539 inhibits Menin–MLL interaction, reducing viability and downregulating MEIS1, FLT3, MEF2C, CDK6, and BCL2 while promoting myeloid differentiation; the Phase 1/2 KOMET-1 trial (NCT04067336) in AML patients, including combination with FLT3 inhibitors, has reported complete responses in two out of six R/R AML patients [43,141]. BMF-219, an orally available irreversible covalent Menin inhibitor, is under Phase I trial (NCT05153330) and downregulates HOX genes and MEIS1, inducing tumor regression (141). SNDX-5613 (FDA-approved) suppresses Menin–MLL interaction, demonstrating a 29% response rate in NPM1-mutated AML patients in the AUGMENT-101 trial (NCT04065399) with RNA-seq confirming HOXA9 and MEIS1 downregulation and upregulation of differentiation markers CD11b, CD14, and CD13 (140). DSP-5336, a selective oral Menin inhibitor, is currently under Phase 1/II evaluation for R/R AML (NCT04988555), with results anticipated in 2030 [42].

DOT1L interacts with the Menin–MLL fusion complex, serving an oncogenic role independent of its catalytic function [44,46]. Menin and DOT1L operate at distinct but interconnected nodes within the same pathogenic pathway: Menin is required for tethering the MLL-fusion complex to chromatin, while DOT1L serves as a downstream effector sustaining aberrant transcription through both enzymatic (H3K79 methylation) and non-enzymatic (scaffolding) mechanisms [44]. This mechanistic complementarity provides a strong rationale for combining Menin inhibition with DOT1L degradation. Preclinical studies have established that Menin inhibitors reduce HOXA9/MEIS1 expression and induce differentiation in *KMT2A*-r leukemia models [48]. Separately, DOT1L degraders have demonstrated potent antileukemic activity and complete tumor regression in orthotopic models [78]. However, direct combination studies evaluating concurrent Menin inhibition and DOT1L degradation have not yet been published. Emerging evidence suggests that Menin inhibition may reduce H3K79me2 levels through altered DOT1L recruitment [44], potentially creating a synthetic lethal dependency that could be exploited by combined approaches. The rationale of combining Menin inhibition with DOT1L degradation offers several theoretical advantages: (1) dual targeting of the MLL fusion complex at both the chromatin-tethering (Menin) and effector (DOT1L) nodes; (2) potential for additive or synergistic effects on leukemogenic gene expression; (3) prevention of resistance emergence through pathway redundancy; and (4) activity against cells that may have developed resistance to either single agent.

Despite the compelling rationale, several limitations must be acknowledged. First, no direct preclinical evidence of synergy between Menin inhibitors and DOT1L degraders has been reported to date. Second, overlapping toxicity profiles (e.g., myelosuppression) may limit the therapeutic window of combination therapy. Third, the optimal sequencing and dosing schedule (concurrent vs. sequential) remains undefined. Fourth, the combination may be effective only in a subset of *KMT2A*-r leukemias that retain dependency on both pathways. Fifth, Menin inhibitors are currently available in oral formulations while DOT1L degraders are administered intravenously, potentially complicating clinical combination regimens. Finally, the cost and complexity of dual therapy must be considered for clinical translation. In future research, rigorous preclinical studies evaluating the combination of Menin inhibitors with DOT1L degraders are urgently needed before clinical translation. Key experiments should assess: (1) synergistic vs. additive effects in multiple *KMT2A*-r leukemia models with diverse fusion partners; (2) impact on resistance emergence; (3) optimal sequencing and dosing; (4) toxicity profiles in combination; and (5) biomarkers to identify patients most likely to benefit. We propose that this combination strategy, while mechanistically compelling, should be approached with caution until these foundational studies are completed.

## 8. Conclusions and Future Perspectives

*KMT2A*-rearranged leukemias remain among the most aggressive and therapeutically challenging hematologic malignances, driven by aberrant recruitment of DOT1L to MLL fusion complexes and persistent activation of leukemogenic transcriptional programs. Although DOT1L has long been recognized as a compelling therapeutic target, clinical experience with catalytic inhibition has revealed the limitations of this approach. Pinometostat provided critical proof-of-concept, yet its modest clinical activity highlighted the inability of catalytic inhibitors to suppress DOT1L’s methyltransferase-independent functions, in addition to pharmacokinetic challenges. The emerging recognition that DOT1L contributes to leukemogenesis not only through H3K79 methylation but also through broader chromatin regulatory and scaffolding functions has significantly reshaped the therapeutic landscape.

Regarding the future of DOT1L catalytic inhibitors, pinometostat (EPZ-5676) remains the only inhibitor to have completed Phase 1 trials. Despite modest single-agent activity, catalytic inhibitors are still being explored in combination regimens, including Phase Ib/II trials with azacitidine (NCT03701295) and standard chemotherapy (NCT03724084). However, preclinical evidence indicates that EPZ-5676 does not enhance azacitidine efficacy, and the persistence of DOT1L’s methyltransferase-independent scaffolding functions remains a fundamental limitation. Consequently, the field has increasingly shifted toward targeted protein degradation to overcome these mechanistic constraints.

These mechanistic insights provide a strong rationale for targeted protein degradation as a next-generation strategy for therapeutic intervention in *KMT2A*-r leukemia. By eliminating the DOT1L protein itself, PROTAC-based degraders offer the opportunity to abrogate both its catalytic and non-catalytic activities, thereby overcoming a major limitation of active-site inhibitors. Concurrently, the field of targeted protein degradation has made remarkable advances in hematologic malignancies, with PROTACs and molecular glues successfully developed against BTK (NX-2127, NX-5948, HSK29116), BCL-XL (DT2216), IKZF1 (CC-220, CC-92480), and GSPT1 (CC-90009) advancing to clinical trials for B-cell malignancies, multiple myeloma, and AML. In this context, the development of VHL-recruiting DOT1L degraders, including MS2133, DOT1L705, and DOT1L808, represents an important advance. DOT1L808 has demonstrated potent and selective degradation, favorable pharmacokinetic properties, robust antileukemic activity in vitro, and complete tumor regression in orthotopic leukemia models without overt toxicity. Despite the mechanistic advantages of PROTACs, their clinical translation faces substantial challenges that are well-documented in the literature [142,143,144]. PROTACs typically possess high molecular weights (>700 Da) and poor membrane permeability, placing them in the ‘beyond-rule-of-5’ (bRo5) chemical space, which limits oral bioavailability and systemic exposure. Suboptimal pharmacokinetic properties, including rapid clearance and metabolic instability, further restrict their clinical applicability. Additionally, the lack of tumor specificity can lead to on-target, off-tumor toxicities and a narrow therapeutic window [145,146,147,148]. Emerging resistance mechanisms, including alterations in E3 ligase function and proteasome activity, represent another concern [149,150]. While innovative strategies such as nanotechnology-enabled delivery systems are being explored to overcome these barriers, the clinical translation of DOT1L-specific PROTACs remains in its early stages. Collectively, these findings establish targeted DOT1L degradation as a promising therapeutic paradigm in the treatment of *KMT2A*-r leukemias, while acknowledging that substantial challenges in pharmacokinetics, toxicity, and clinical validation remain to be addressed before this approach can reach patients.

## Figures and Tables

**Table 2:** Evidence for *DOT1L* methyltransferase-independent functions: model systems and leukemia relevance.

Model System	Key Finding	Reference	Established in *KMT2A*-r Leukemia?	Leukemia Relevance	Validation Status
*KMT2A*-r Leukemia	Genetic depletion produces greater effects than catalytic inactivation	Cao et al. (2020) [35]	Yes	Directly establishes non-catalytic functions in leukemia	Established
Erythroleukemia	DOT1L regulates transcription initiation independent of catalytic function	Wu et al. (2021) [73]	No	Mechanistically relevant but requires direct validation	Requires validation
Embryonic Stem Cells	DOT1L functions as histone H1 chaperone with NPM1	Zhao et al. (2023) [101]	No	Supports moonlighting concept; leukemia relevance uncertain	Hypothesis
Embryonic Stem Cells	DOT1L regulates primitive erythropoiesis independent of catalysis	Malcom et al. (2021) [100]	No	Developmental functions may not translate to leukemia	Hypothesis
Yeast	Dot1 promotes H2B ubiquitination independent of H3K79 methylation	Van Welsem et al. (2018) [102]	No	Mechanistic insight; mammalian relevance uncertain	Mechanistic insight
Yeast	Dot1 functions as histone chaperone	Lee et al. (2018) [103]	No	Mechanistic insight; mammalian relevance uncertain	Mechanistic insight

**Table 3:** DOT1L histone methyltransferase inhibitors structures, functions, and development approaches.

Inhibitors	DOT1L (IC50)	Function	Critical Assessment/Limitations	Refs
EPZ4777	0.3 nM	EPZ004777, a potent and selective inhibitor of DOT1L. In MLL cells, it selectively impedes H3K79 methylation and disrupts the expression of leukemogenic genes. The compound induces selective cell death in leukemic cells carrying the MLL gene translocation, sparing non-MLL-translocated cells. In vivo administration of EPZ004777 in a mouse MLL xenograft model extends survival, highlighting DOT1L inhibition as a promising strategy for targeted therapeutics against MLL.	Established proof-of-concept; poor drug-like properties (size, hydrophobicity); limited in vivo efficacy	[3]
EPZ5676	0.08 nM	EPZ-5676, a highly selective inhibitor of DOT1L histone methyltransferase, extends 37,000-fold over other methyltransferases, ensuring specificity in its action and exhibits potent action against *KMT2A*-rearranged leukemia, characterized by remarkable specificity, H3K79 methylation inhibition, and significant tumor regression in preclinical models, warranting clinical investigation.	Advanced to clinical trials; modest clinical efficacy despite potency; continuous IV infusion required; non-catalytic functions remain intact.	[4]
Compound 10	0.0001 nM	Modifying compound 9 by replacing one methoxy group with an ionizable piperazine conferred a log D decrease superior to 1.4 and a further increase in potency. Compound 10 showed slow kinetics (t1/2 > 5 h), but it showed no inhibition below 5 μM. Dosage 300 mg/kg reaching to 4 to 8 μM in blood over 24 h after a single administration and no loss of exposure after multiple doses but rather accumulation. Unfortunately compound not tolerated at such high doses.	Exceptional potency but poor tolerability at required high doses; limited clinical applicability.	[108]
Compound 10	234 nM	Compounds 10 and 11 mimic EPZ5676’s action but offer enhanced mouse administration options (oral and intraperitoneal routes) and display promising antileukemic properties in preclinical PDX leukemia models. This accessibility now allows for assessing single and combined treatment regimens of DOT1L inhibitors with other drugs in relevant in vivo systems, creating novel therapeutic possibilities.	Improved administration but reduced potency; potential for combination studies.	[69]
Compound 11	330.7 nM	Compounds 10 and 11 mimic EPZ5676’s action but offer enhanced mouse administration options (oral and intraperitoneal routes) and display promising antileukemic properties in preclinical PDX leukemia models. This accessibility now allows for assessing single and combined treatment regimens of DOT1L inhibitors with other drugs in relevant in vivo systems, creating novel therapeutic possibilities.	Similar limitations to Compound 10; reduced potency compared to EPZ5676.	[69]
SGC0946	0.3 nM	It is a brominated analog of EPZ004777 and a more potent DOTL1 inhibitor than EPZ004777. Though this brominated analogue shows no effect on selectivity, reduced the level of methylation more potently than EPZ004777. In MLL-AF9 fusion oncogene-based experimental leukemia model, SGC0946 showed a selective decrease in cell survival with a significant decrease in MLL target genes (HOXA9 and MEIS1)	More potent than EPZ004777; no improved selectivity; effects on MLL target genes confirmed.	[105]
SGC0947	3.5 nM	SGC0947 exhibits a tenfold increase in IC50 compared to EPZ4777, indicating a notable contribution of this group to the interaction. In MV4;11 leukemia cells expressing an MLL/AF4 fusion protein, depletion of H3K79me2 was observed at 48 h with SGC0947.	Confirms structural requirements for potency; less clinically relevant.	[105]
DC_L115	1.5 μM	DC_L115, a potent inhibitor, demonstrates significant inhibitory activity against DOT1L with an IC50 of 1.5 μM. Utilizing surface plasmon resonance-based binding assays, DC_L115 exhibits a binding affinity of 0.6 μM to DOT1L in vitro. Additionally, it selectively hinders the proliferation of *KMT2A*-rearranged cells, showcasing an IC50 value of 37.1 μM.	Selectively cytotoxic to *KM2TA*-r cells; moderate potency limits clinical potential.	[109]
FED1	2.9 nM	An adenine derivative and analog of EPZ004777 where at position 7 carbon is replaced by adenine nitrogen shows potent inhibitory activity for DOT1L.	Potent but limited structural diversity; binding mode not fully characterized.	[105]
FED2	40.3 nM	A derivative of FED1 where t-butyl was replaced by a methyl group shows a reduction in biochemical activity and confirm the role of t-butyl group in the binding affinity.	Reduced potency confirms t-butyl group importance; limited clinical potential.	[105]
Selenopsammaplin	0.06 μM	Selenopsammaplin A and its thirteen analogs were developed as DOTL1 inhibitors, and the majority of them show cytotoxicity against cancer cells and strong inhibitory activity toward *DOT1L* for antitumor potential. Selenopsammaplin A was newly designed and synthesized with a 25 percent overall yield in eight steps from 3-bromo-4-hydroxybenzaldahyde.	Novel scaffold; anticancer activity in breast cancer; limited leukemia data.	[110]
Massonianoside B (MA)	0.40 μM	MA is a potent SAM-competitive inhibitor of DOT1L. MA selectively inhibits proliferation and causes apoptosis in *KMT2A*-rearranged leukemia cells by inhibiting H3K79 methylation and suppresses MLL fusion target gene expression.	SAM-competitive; selective for *KMT2A*-r cells; limited in vivo data.	[111]
Compound 25	1 μM	Compound 25 stands out as the most potent inhibitor of DOT1L in the novel series, displaying an impressive IC50 of 1.0 μM—a notable 40-fold improvement compared to hit 9. Its efficacy is underscored by significant on-target effects observed in a *DOT1L*-dependent murine cell line.	40-fold improvement over hit; on-target effects confirmed; limited potency.	[112]
MU1656	2 nM	MU1656, a carbocyclic C-nucleoside analog of the natural nucleoside derivative EPZ004777 and the clinical candidate EPZ5676 (pinometostat), exhibits potent and selective inhibition of DOT1L both in vitro and in cells. Notably, the most potent compound, MU1656, demonstrates enhanced metabolic stability and markedly reduced in vivo toxicity compared to pinometostat.	Enhanced metabolic stability; reduced toxicity compared to pinometostat; no clinical data.	[113]
Compound 3	20.94 μM	Compound 3 emerges as a novel DOT1L inhibitor with an in vitro IC50 value of 20 μM. Furthermore, it effectively hampers the proliferation of *KMT2A*-rearranged leukemia cells, such as MV4-11, inducing cell cycle arrest and apoptosis.	Early-stage discovery; limited potency; in vitro effects confirmed.	[114]
Compound 6	8.29 μM	Compound 6 as a novel DOT1L inhibitor with a scaffold of [1,2,4]-triazolo-[3,4-b] [1,3,4]-thiadiazole, which specifically inhibits proliferation and induces apoptosis of *KMT2A*-r leukemic cell lines with an IC50 value of 21.9 μM	Novel scaffold but weak potency; limited therapeutic utility.	[117]

**Table 4:** Clinical trial data for DOT1L inhibitor EPZ-5676 in leukemia.

Clinical Trial ID	Drug	Phase	Objectives	Patient Population	Number of Patients	Treatment Protocol	Biomarkers Assessed
NCT01684150	EPZ-5676	Phase I	Safety, Tolerability, PK and PD	Relapsed/Refractory *KMT2A*-rearranged leukemias and advanced hematological malignancies	51	EPZ-5676 at doses of 54 and 90 mg/m^2^/day via continuous intravenous infusion in 28-day cycles. Adverse events: fatigue (39%), nausea (39%), constipation (35%), febrile neutropenia (35%). Notably, two patients achieved complete remission at 54 mg/m^2^/day.	H3K79me2 levels in peripheral blasts; PK/PD correlation
NCT02141828	EPZ-5676	Phase I	Efficacy, Dose Escalation and Response Rates	*KMT2A*-rearranged leukemias	18	EPZ-5676 demonstrated safe profile, RP2D of 70 mg/m^2^ CIV for children > 1 year old. Temporary reductions in blasts in 40% of patients, no objective responses noted. Further exploration of pinometostat combinations warranted.	Plasma PK; blast H3K79me2
NCT03701295	EPZ-5676	Phase II and Phase I	Tolerability and Efficacy	Relapsed, Refractory, or Newly Diagnosed *KMT2A*-rearranged AMLs	36	EPZ-5676 via intravenous infusion continuously (days 1–28) + azacytidine intravenously or subcutaneously for 7 of first 10 days. Treatment repeated every 28 days for six cycles, unless progression or unacceptable toxicity.	Not specified
NCT03724084	EPZ5676 + Standard chemotherapy	Phase I and II	Tolerability and Response Rate	Newly *KMT2A*-Rearranged AMLs	6	Currently active but not enrolling new participants.	Not specified

**Table 5:** Clinical trials of PROTACs in hematological malignancies.

Clinical Trial Name	PROTAC Name	Target Proteins	Indication	Trial Identifier
NX-2127 Phase 1a	NX-2127	BTK	Relapsed or Refractory B Cell Malignancies	NCT04830137
NX-5948 Phase 1a/1b	NX-5948	BTK	Relapsed and Refractory B-cell Malignancies	NCT05131022
HSK29116 Phase 1a/1b	HSK29116	BTK	Relapsed or Refractory B Cell Malignancies	NCT04861779
BGB-16673 Phase 1	BGB-16673	BTK	B Cell Malignancies	NCT05006716
DT2216 Phase 1	DT2216	BCL-XL	Relapsed and Refractory Hematological Malignancies	NCT04886622
CC-220-Phase 1b/2	CC-220	IKZF1	Multiple Myeloma, Diffuse Large B-cell Lymphoma	NCT02773030
CC-92480 Phase 1/2	CC-92480	CRBN	Relapsed and Refractory Multiple Myeloma	NCT03989414
CC-90009 Phase 1	CC-90009	GSPT1	Acute Myeloid Leukemia (AML), Refractory AML, MDS	NCT02848001 /NCT04336982
CC-99282 Phase 1	CC-99282	IKZF1, IKZF3	Relapsed and Refractory CLL, R/R NHL	NCT04434196 /NCT03930953
CFT7455 Phase 1/2	CFT7455	IKZF1, IKZF3	Relapsed and Refractory Multiple Myeloma, Non-Hodgkin’s Lymphomas	NCT04756726

**Table 6:** A balanced comparison of all three DOT1L degraders.

Feature	MS2133 (Yim et al.)	DOT1L705 (Quan et al.)	DOT1L808 (Quan et al.)
E3 Ligase Recruited	VHL	VHL	VHL
DC50	Not reported	0.33 μM	5 nM
Dmax (Maximum Degradation)	~90%	~90%	>95%
Degradation Kinetics	Concentration and time-dependent	Time-dependent	Time-dependent
Selectivity	Selective for DOT1L over other methyltransferases	Selective for DOT1L	Selective for DOT1L
Effect on mRNA Expression	No effect on DOT1L mRNA	Not reported	Not reported
In Vitro Anti-proliferative Activity	Yes (*KMT2A*-r leukemia cells)	Yes (*KMT2A*-r leukemia cells)	Yes (*KMT2A*-r leukemia cells)
Activity in Menin Inhibitor-Resistant Cells	Not reported	Yes	Not reported
In Vivo Efficacy	Not reported	Not reported	Complete tumor regression in orthotopic model
Pharmacokinetic Properties	Not reported	Moderate	Favorable
Toxicity Profile	No toxicity to normal cells	Not reported	No overt toxicity in mice
Developmental Stage	Preclinical (proof-of-concept)	Preclinical	Preclinical (most advanced in vivo)
Advantages	First reported DOT1L degrader; establishes proof-of-concept	Activity in Menin inhibitor-resistant cells	Potent degradation; in vivo efficacy; favorable PK
Limitations	No in vivo or PK studies reported	Moderate potency; no in vivo data	Longer-term toxicity studies needed

**Table 7:** Menin inhibitors in clinical trials for *KMT2A*-rearranged leukemias.

Clinical Trial Details	Drugs	Trial Phase	Objectives	Patient Populations	Key Findings/Results
NCT04752163	DS-1594a	Phase I	Safety, Dosing, Efficacy	AML, ALL, CML, MDS	In progress, Results expected.
NCT04067336	KO-539	Phase I/II	Safety, Dosing, Efficacy	AML, ALL, Mixed Lineage Leukemia, NPM1-m AML	Recruiting, in progress, results expected.
NCT05153330	BMF-219	Phase I	Safety, Dosing, Efficacy	AML, ALL, DLBCL, MM, CLL	Terminated
NCT04065399	SNDX-5613 (FDA-Approved)	Phase I/II	Safety, Dosing, Efficacy	AML, ALL, Mixed Lineage Acute Leukemia	Recruiting, in progress, results expected.
NCT04988555	DSP-5336	Phase I/II	Safety, Dosing, Efficacy	AML, ALL	Recruiting, in progress, results expected.

## Data Availability

No new data were created or analyzed in this study. Data sharing is not applicable to this article.

## Abbreviations

The following abbreviations are used in this manuscript:

AEP	AF4 family/ENL family/P-TEFb
ASH1L	(histone-lysine N-methyltransferase)
AFF1	AF4/FMR2 family member 1
AFF4	AF4/FMR2 family member 4
AF10	ALL-1 Fused Gene from Chromosome 10
AML	Acute Myeloid Leukemia
AF4	ALL-1 Fused Gene from Chromosome 4
AF6	ALL-1 Fused Gene from Chromosome 6
AF9	ALL-1 Fused Gene from Chromosome 9
BET	Bromodomain and Extra Terminal Domain
B-ALL	B-lineage Acute Lymphoblastic Leukemia
CDK9	Cyclin-Dependent Kinase 9
CLL	Chronic Lymphoblastic Leukemia
CNS	Central Nervous System
CTD	C-Terminal Domain
CYP33	Cyclophilin
DotCom	DOT1L-Containing Complex
DOT1L	Disruptor of Telomeric Silencing 1-Like
DNMT1	(DNA methyltransferase 1)
EAPs	ENL-Associated Proteins
E1A	Early region 1A
EAFs	ELL associated proteins
ENL	Eleven-Nineteen Lysine-Rich Leukemia
ESCs	Embryonic stem cells
EZH2	Enhancer of Zeste Homolog 2
ELL	Eleven-Nineteen Leukemia
H3K79	Histone H3 Lysine 79
H3K79me1	Histone 3 Lysine 79 Monomethylation
H3K79me2	Histone 3 Lysine 79 Dimethylation
H3K79me3	Histone 3 Lysine 79 Trimethylation
HDACs	histone deacetylases (HDACs
HTS	High-Throughput Screening
MM	Multiple Myeloma
HOX	Homeobox genes
MLL	Mixed-Lineage Leukemia
MEIS1	Meis homeobox 1
MBD1	methyl-CpG-binding protein 1 (MBD1)
NPM1	Nucleophosmin
P-TEFb	Positive Transcription Elongation Factor b
PRMT5	Protein Arginine Methyltransferase 5
PHD domains	Plant homeodomain
PD	Pharmacodynamics
PK	Pharmacokinetics
PROTACs	Proteolysis-Targeting Chimeras
POI	Protein of Interest
RNA Pol II	RNA Polymerase II
SAM	(S)-adenosyl-l-methionine
SAH	(S)-adenosyl-l-homocysteine
SEC	Super Elongation Complex
TPD	Targeted Protein Degradation
TPET	Targeted Protein Elimination Technology
YEATS	Yaf9, ENL, AF9, Taf14, and Sas5
